# Consistency and Diversity of Spike Dynamics in the Neurons of Bed Nucleus of Stria Terminalis of the Rat: A Dynamic Clamp Study

**DOI:** 10.1371/journal.pone.0011920

**Published:** 2010-08-03

**Authors:** Attila Szücs, Fulvia Berton, Thomas Nowotny, Pietro Sanna, Walter Francesconi

**Affiliations:** 1 BioCircuits Institute, University of California San Diego, La Jolla, California, United States of America; 2 Molecular and Integrative Neurosciences Department, The Scripps Research Institute, La Jolla, California, United States of America; 3 Centre for Computational Neuroscience and Robotics, School of Informatics, University of Sussex, Brighton, United Kingdom; 4 Department of Experimental Zoology, Balaton Limnological Research Institute of the Hungarian Academy of Sciences, Tihany, Hungary; 5 Department of Biology, University of Pisa, Pisa, Italy; Mount Sinai School of Medicine, United States of America

## Abstract

Neurons display a high degree of variability and diversity in the expression and regulation of their voltage-dependent ionic channels. Under low level of synaptic background a number of physiologically distinct cell types can be identified in most brain areas that display different responses to standard forms of intracellular current stimulation. Nevertheless, it is not well understood how biophysically different neurons process synaptic inputs in natural conditions, i.e., when experiencing intense synaptic bombardment *in vivo*. While distinct cell types might process synaptic inputs into different patterns of action potentials representing specific “motifs” of network activity, standard methods of electrophysiology are not well suited to resolve such questions. In the current paper we performed dynamic clamp experiments with simulated synaptic inputs that were presented to three types of neurons in the juxtacapsular bed nucleus of stria terminalis (jcBNST) of the rat. Our analysis on the temporal structure of firing showed that the three types of jcBNST neurons did not produce qualitatively different spike responses under identical patterns of input. However, we observed consistent, cell type dependent variations in the fine structure of firing, at the level of single spikes. At the millisecond resolution structure of firing we found high degree of diversity across the entire spectrum of neurons irrespective of their type. Additionally, we identified a new cell type with intrinsic oscillatory properties that produced a rhythmic and regular firing under synaptic stimulation that distinguishes it from the previously described jcBNST cell types. Our findings suggest a sophisticated, cell type dependent regulation of spike dynamics of neurons when experiencing a complex synaptic background. The high degree of their dynamical diversity has implications to their cooperative dynamics and synchronization.

## Introduction

The biophysical mechanisms underlying the conversion of synaptic inputs into action potentials have been subject of intense experimental and theoretical research [1], [2], [3]. Neurons show a remarkable degree of computational complexity that is the result of the differential activation and inactivation of their voltage-gated membrane conductances during the integration of synaptic inputs. The multitude of the voltage-gated ionic channels suggest that they all make an important contribution to the firing pattern of neurons. Indeed, physiologically distinct cell types can readily be identified in most brain areas that display different voltage responses when stimulated with rectangular current waveforms. The temporal structure of firing in response to depolarizing current pulses is commonly used for their categorization [4], [5]. Additionally, sag-responses and rectification during constant hyperpolarizing current or post-inhibitory rebound are hallmarks of specific membrane conductances that can be used for physiological classification of neurons. Nevertheless, accumulating evidence suggests that boundaries between physiologically distinct cell types might be less clear than usually believed. For instance, cortical neurons in vivo conditions have been shown to display richer dynamics and a wider repertoire of firing patterns than when studied in slice preparations where neurons usually experience sparse synaptic inputs [5]. It is therefore an important and challenging problem of neurophysiology to understand how biophysically different types of neurons function in a complex synaptic environment such as that in the functioning brain. If synaptic inputs arrive synchronously to distinct populations of postsynaptic neurons, how different spike responses will they produce? How this will affect the synchronization of microcircuits and the transfer of temporally precise firing patterns? Clearly, the biophysical variability that is observed in different cell types presents the possibility that the output of component neurons might represent different motifs of network activity when receiving synchronous synaptic inputs [6]. Nevertheless, physiologically distinct cell types as defined by conventional methods in in vitro preparations might appear less different when experiencing intense synaptic bombardment and operating in the high conductance state [7].

In the present paper we used synthetic synaptic inputs introduced with dynamic clamp to stimulate biophysically distinct types of neurons in a brain slice preparation. The bed nucleus of stria terminalis (BNST), the subject of our investigation, is a brain area that plays an important role in the regulation of stress and reward. The BNST is part of the extended amygdala, an anatomical macrostructure that comprises several basal forebrain structures to form a grey matter continuum sharing similarities in morphology, neurochemistry and connectivity [8], [9]. Drugs of abuse and stress have been shown to produce changes in synaptic and non-synaptic forms of neural plasticity in the BNST [10], [11]. The juxtacapsular BNST (jcBNST) is a small nucleus in the dorsolateral BNST that has direct projections to the medial part of the central nucleus of the amygdala (CEAm) and can indirectly also influence the CEA through its projections to the basolateral amygdala (BLA) and other cell groups that in turn send projections to the CEA [12]. Thus, changes in the computational properties of jcBNST neurons may contribute to the overall amygdala output [13].

The jcBNST contains three types of physiologically different GABAergic interneurons. These cell types have different amounts of specific voltage-gated membrane conductances such as the hyperpolarization-activated nonspecific inward current, the transient K- current or the low-threshold Ca-current [14]. We sought to determine if these neurons produce different spike responses - firing signatures - to the same pattern of simulated random synaptic inputs under dynamic clamp conditions. Somewhat unexpectedly, the three types of jcBNST neurons did not display marked differences in their spike responses under such conditions. However, our experiments revealed remarkable, cell type dependent behavior at the level of single spikes and their timing precision. In this respect, biophysical variability and diversity might have a stronger impact on the millisecond-resolution temporal structure of postsynaptic spike trains than on their dynamics at longer time scales.

## Results

### General physiological properties of jcBNST neurons

**Figure 1:**
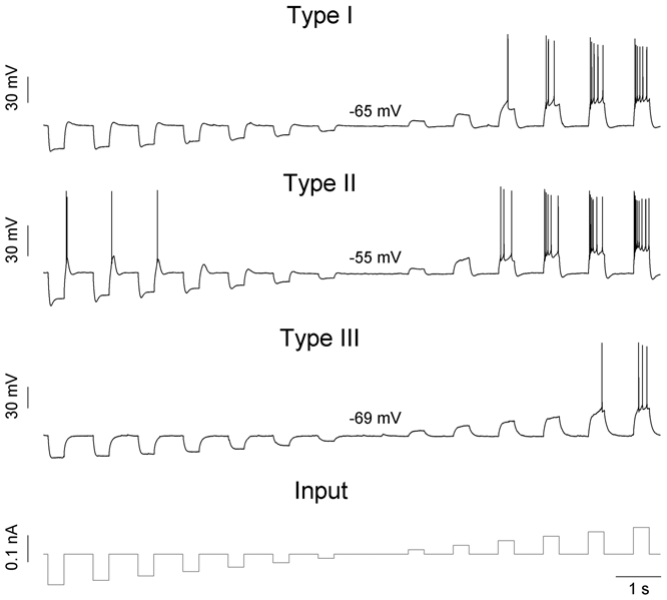
DC current stimulation reveals three physiologically different types of jcBNST neurons. The bottom trace shows the injected current waveform (350 ms steps, current level incremented by 20 pA). The type I neuron on top shows a depolarizing sag during hyperpolarizing steps but no post-inhibitory rebound firing (PIR). The type II neuron in the middle displays a larger depolarizing sag and robust PIR following more hyperpolarized levels of the membrane potential. The type III neuron lacks the sag-response visible in the previous traces and starts firing at higher levels of depolarizing current than the others. Resting membrane potential values were as shown above each trace.

To identify the physiological type of jcBNST neurons first we performed experiments with standard protocols of intracellular current injection [14]. Specifically, we used rectangular current pulses of both hyperpolarizing and depolarizing polarity and observed the neurons voltage responses. According to this protocol, three cell types can be distinguished in the rat jcBNST, as previously noted [11], and consistent with a previous description in the rat anterolateral BNST as a whole [14] (Fig. 1). The 3 cell types display characteristic differences in their voltage responses and these visual features indicate the presence and amount of specific voltage-gated ionic conductances in their membrane. Specifically, type I neurons were characterized by the presence of a depolarizing sag in response to hyperpolarizing current injection indicative of activation of I_h_, and the absence of rebound firing after release from hyperpolarization (Fig. 1, Type I). In all, 25 type I neurons were used in our experiments, first subjected to rectangular current stimulation and then dynamic clamp stimulation. Type II neurons (n = 34) had a larger depolarizing sag, indicative of a stronger I_h_, and they also displayed robust post-inhibitory rebound firing (Fig. 1, Type II) partly caused by the activation of the low-threshold Ca-current (I_T_). Type III neurons (n = 35) did not have either a depolarizing sag or rebound firing, but exhibited rectification with hyperpolarizing current injection (Fig. 1, Type III). When the three types of neurons received suprathreshold depolarizing current injection, they displayed firing patterns that were also distinguishable. Specifically, type I neurons exhibited a regular firing pattern with moderate spike frequency adaptation. Conversely, responses of type II neurons under DC depolarizing current injection usually showed a depolarizing “hump” shortly after the onset of current injection resulting in an initial burst that developed into more regular firing. Type III neurons exhibited a depolarizing ramp and delayed firing under DC step depolarization and their firing patterns showed an accelerating behavior unlike the other two types of neurons. Importantly, type III neurons displayed more hyperpolarized resting membrane potentials than the other two types of neurons (Table 1.). Furthermore, these neurons had lower input resistance and required stronger depolarizing current to fire than the other two types. Type O neurons represent a new class and they are described later.

**Table 1:** Physiological properties of four types of jcBNST neurons (numbers indicated in parentheses).

	Resting V_m_ [mV]	Resistance [MΩ]	Reliability [%]	Precision [ms]
Type I (7)	−59.4±7.5	258±59	86.2±8.2	0.80±0.28
Type II (8)	−61.0±6.7	329±97	88.04±7.5	0.83±0.18
Type III (8)	−72.9±5.1*#	171±72*#	90.5±4.0	0.92±0.21
Type O (4)	−68.5±9.1	272±121	71.0±11.8 †	1.25±0.36

The input resistance of the neurons was measured using −100 pA hyperpolarizing current and by seeking the local minimum of the membrane potential. The reliability and precision parameters are means (± S.D.) calculated from responses containing in average 20 spikes per trial under the 30 Hz tonic protocol. Average spike jitter is below 1 ms for all three cell types under dynamic clamp stimulation. Symbols *, # and † indicate significant differences (p<0.05) from the corresponding values of type I, II and III neurons, respectively.

### Reliability and precision of spike timing in three types of jcBNST neurons

Synaptically isolated jcBNST neurons were at rest with no sign of subthreshold oscillations or slow modulations in their membrane potential. To induce temporally complex, in vivo-like firing in these neurons, we subjected them to a barrage of stochastic, computer-generated synaptic inputs (frozen noise) via dynamic clamp. The noisy input consisted of an excitatory and an inhibitory presynaptic waveform, two trains of artificial spikes both having a mean firing rate of 30 Hz and Gaussian distributed interspike intervals with a standard deviation of 25 ms. This stimulation proved to be efficient to induce vigorous and complex firing patterns in the jcBNST neurons so they visited a wide dynamical range of their activity space. At the same time, the impact of individual EPSPs and IPSPs on the postsynaptic firing could be accurately evaluated, because, on average, there was a 15 ms separation between consecutive synaptic events.

**Figure 2:**
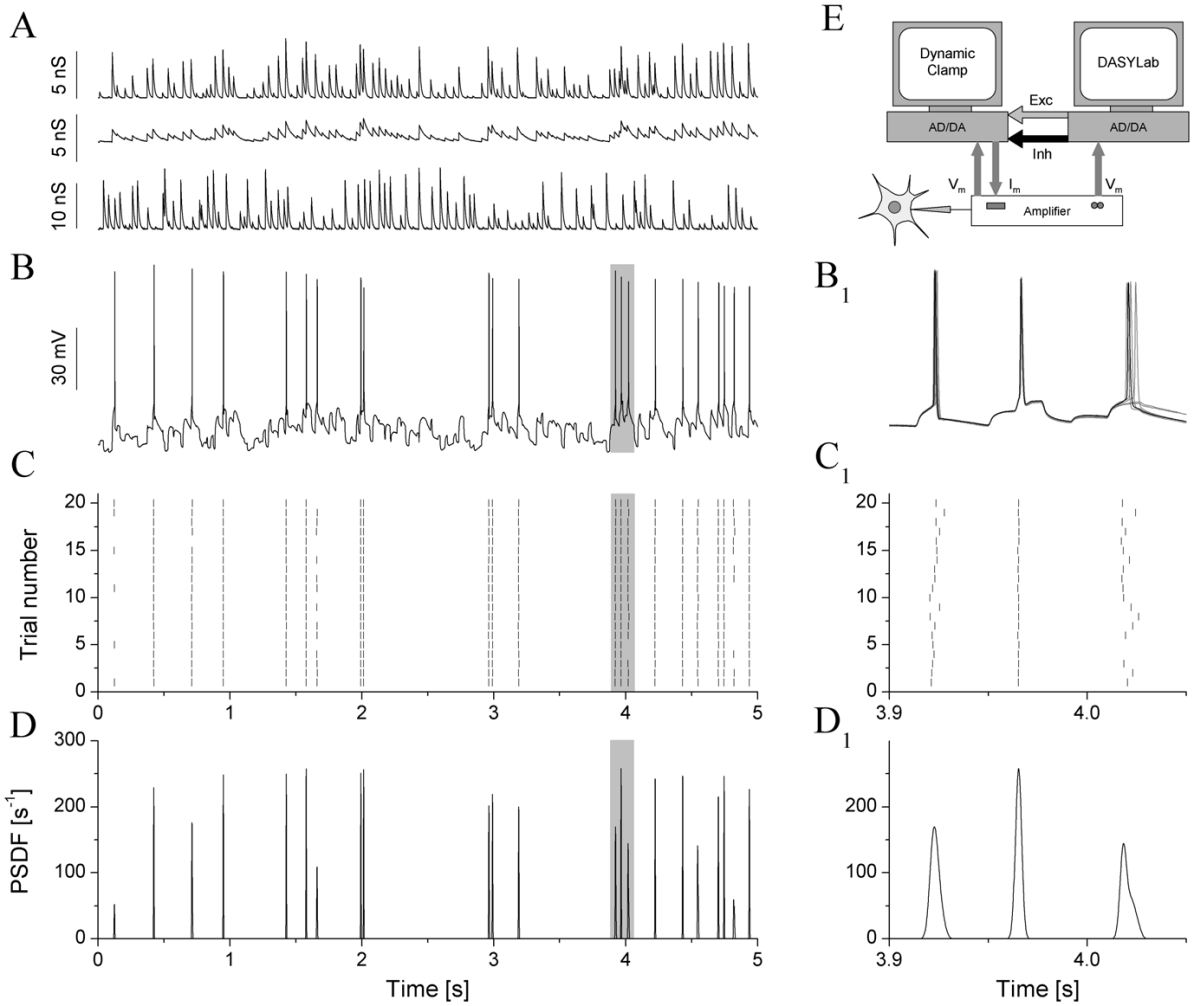
jcBNST neurons display highly reproducible spike responses under the action of simulated synaptic inputs. One excitatory and one inhibitory presynaptic voltage waveforms are used to generate a total of three synaptic conductances: 1 fast (AMPA-type), 1 slow (NMDA-) excitatory and 1 fast inhibitory (GABA-) inputs for the jcBNST neuron (A). The type II neuron on B responds with irregular firing when stimulated with the above inputs, but this pattern is very reproducible across trials, as shown by the peri-stimulus scatter plot in C. Peri-stimulus spike density function (PSDF) of the above pattern is shown in D. The right side panels (B_1_, C_1_, D_1_) are zoomed sections of the corresponding graphs (6 overlapping voltage traces shown in B_1_). The third spike event is less reliable and less precise than the preceding two. The schematic of the dynamic clamp system is shown in E.

First we compared spike responses of the 3 jcBNST neuronal types under frozen noise stimulation via dynamic clamp as shown in Fig. 2. As a first observation on the evoked firing patterns, jcBNST neurons, regardless of their physiological type, responded with high reliability and precision under synaptic stimulation via dynamic clamp (Table 1). Peri-stimulus scatter plots of the evoked responses revealed accurate reproduction of spike patterns appearing as vertically aligned spike events in such plots (Fig. 2C). To identify spike events, i.e. spikes that were reliably reproduced in more than 33% of stimulus presentations we used the peri-stimulus spike density function (PSDF, Fig. 2D) as a continuous estimate of firing frequency along the course of the stimulus. Peaks in a PSDF correspond to single spike events and the amplitude of the peaks is positively correlated with both the reliability and precision of firing (Fig. 2B_1_–D_1_). The *mean* reliability and precision are temporal measures of the entire spike response (multiple presentations of the same input) and they are calculated by averaging the reliability and precision values determined for the individual spike events. These measures are pooled and shown for the three different cell types in Table 1. However, both the reliability and precision vary greatly across events even in the same pattern of stimulation, i.e. both very precise and more “jittery” spike events are observed along the stimulus (Fig. 2B_1_–D_1_). Indeed, the precision of spike timing ranged from 0.2 ms to 3 ms in our experiments. As observed frequently and to be shown later, EPSPs barely crossing the spike threshold of the neuron result in less reliable and less precise spikes than strong EPSPs with fast rise times. Hence, the mean values of reliability and spike jitter serve only as gross metrics of the observed spike dynamics and additional parameters are to be used to describe the rich dynamics of neurons under physiologically realistic inputs.

**Figure 3:**
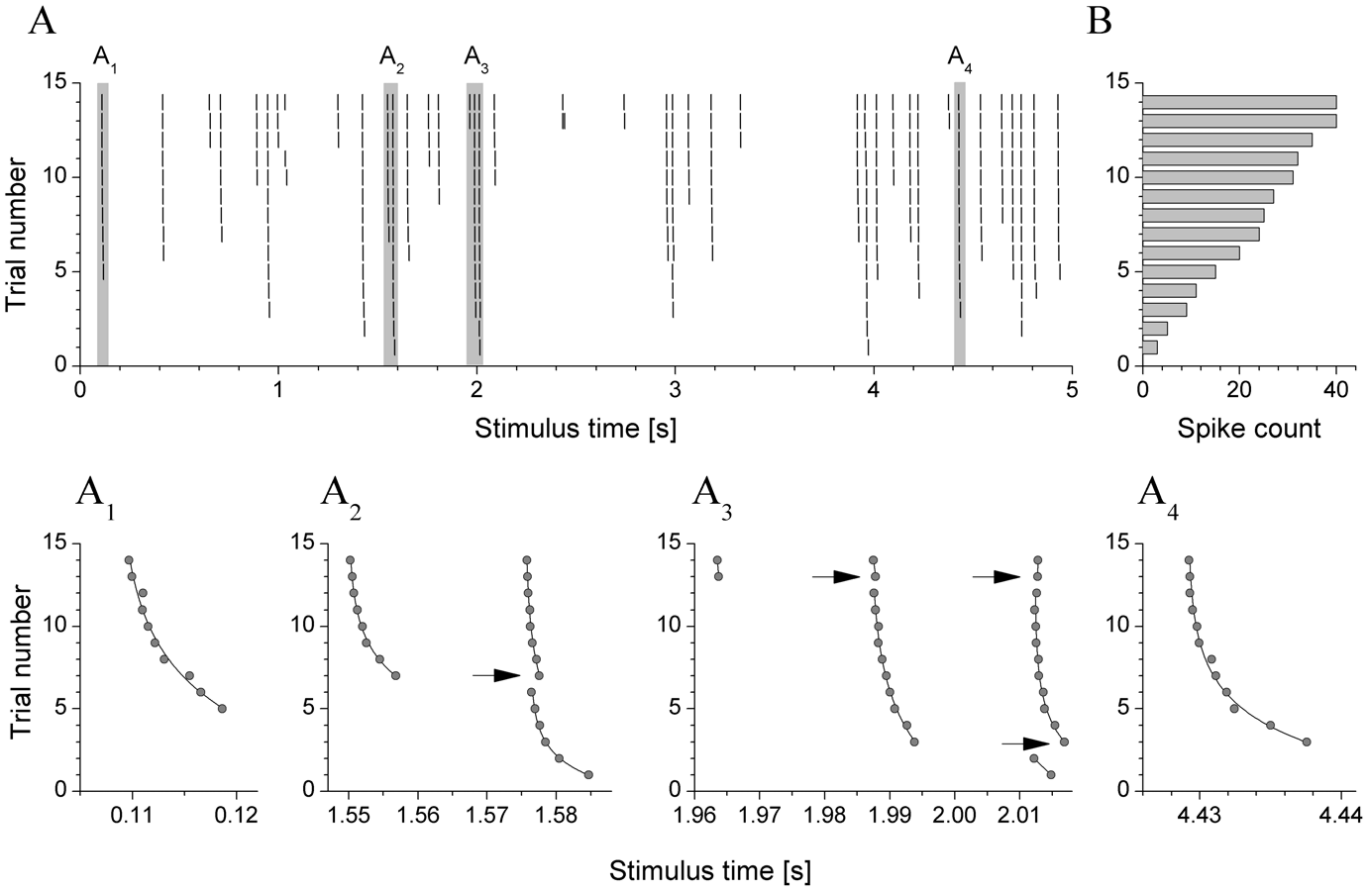
Gradually increasing synaptic inputs leads to an increasing number of spikes and decreasing latency between the pre- and postsynaptic spikes. (A) Peri-stimulus scatter plot of a type II neuron receiving synaptic inputs with increasing maximal conductance (from 5 nS to 18 nS; trial number 1 to 14, respectively). The spike count in the successive trials increases monotonously (B). Four selected sections (gray bars) of the peri-stimulus plot are displayed below. A_1_ is an example of a single “clear” spike with exponentially decreasing latency. A_2_ shows the effect of a new spike in trial #7 which delays the following spike already present in the earlier trials. A_3_ is similar, but here two new spikes appear in trial #3 and #13, respectively; the delaying effect is weaker when the excitatory synaptic conductance is stronger, i.e. at trial #13 (17 nS). A_4_ is another example for a “clear” spike with exponentially decreasing latency (like on panel A_1_).

As noted above, type III neurons were the most hyperpolarized and required stronger depolarization to fire. Hence, a particular set of conductance parameters that was effective in driving vigorous firing in a type I or II neuron was usually ineffective for a type III neuron. However, comparing spike responses of various types of jcBNST neurons required not only that they received the same temporal pattern of synaptic input but also that they fired nearly the same number of spikes during the stimulation (under one sweep of the stochastic input). Hence, we made an effort to keep the spike count constant across different neurons. A target spike count of 20 was used in most experiments meaning that the 5 s stimulus was expected to evoke approximately 20 spikes in each successive trial of the experiment. As shown in the example of Fig. 3, a type II neuron easily produced up to 40 spikes in response to the synaptic stimulation (5 s trials), so a target spike count of half of that offered a good choice. This way we were able to obtain sufficient number of spike responses for statistical evaluation while limiting the risk of degrading the cell due to overstimulation.

As anticipated from their passive electrical properties, different types of neurons required different values of maximal conductances to emit the targeted number of spikes. One way to determine the required parameter settings was to change the 3 maximal conductances manually (but keeping the 1/1/2 ratio) and to observe the spike response in one or two successive trials. The target spike number was typically found after testing 3 or 4 parameter settings. However, in most cases we used a more efficient and systematic method by automatically incrementing the synaptic conductance values in the dynamic clamp (by scripting). Here we set a low initial value for the maximal conductances (e.g. 2/2/4 nS) and increased those in small equal steps in the successive trials. These experiments revealed interesting features of the spike responses and showed how spike timing reliability/precision depended upon the strength of the EPSPs.

### Correlation of latency and precision of spike timing

Figure 3 demonstrates the behavior of a representative neuron in an experiment where gradually increasing conductances were used. The spike count in the successive trials increased progressively with the conductance gain. Initially only 3 spikes were observed while gradually stronger EPSPs resulted in more robust firing (Fig. 3B). Furthermore, spikes that appeared at some time in the experiment remained “in place” in the successive trials i.e. whenever a particular EPSP became suprathreshold, the corresponding postsynaptic spike was reliably emitted in the subsequent trials. Peri-stimulus analysis of the responses also showed that increasingly stronger EPSPs evoke postsynaptic spikes with shorter latency resulting in a slight bending of the individual spike events (tick marks moving to the left) in the peri-stimulus scatter plots (Fig. 3A_1_–A_4_). The spike time vs. maximal conductance relationship could be well fitted by a negative slope monoexponential for each spike event. This behavior was remarkably accurate and consistent among spike events in the entire spectrum of neurons we studied. The slope of the spike count vs. conductance relationship and the shape of the exponentials depended on the particular cell and also on the particular spike event, but the overall behavior was very consistent. Hence, the magnitude of the local EPSC has a strong influence on the timing of the corresponding postsynaptic spike. As the exponential fits show, the latency (interval between the pre- and postsynaptic spike) is determined by the EPSC strength at millisecond precision.

As noted, gradual amplification of the synaptic inputs resulted in increase of the spike count rather than re-patterning of the response. Still, newly arriving spikes imposed a profound effect on the timing of ones already present in the response. For instance, when an EPSP became suprathreshold and a new spike appeared in the spike train, it imposed a shifting effect on an adjacent “old” spike that was already present in the previous trials (Fig. 3A_2_ and A_3_). This effect is very clearly seen when the interval between the new and the old, trailing spike is less than 50 ms. The spike shifting effect results in a discontinuity in the conductance-spike timing relationship, i.e. a sudden jump from the course of the monoexponential (see arrows in Fig. 3A_2_, A_3_). This interference between adjacent spikes becomes more significant when short interspike intervals occur more frequently as observed with increasing firing rates.

### Similarity of spike responses in different types of neurons

**Figure 4:**
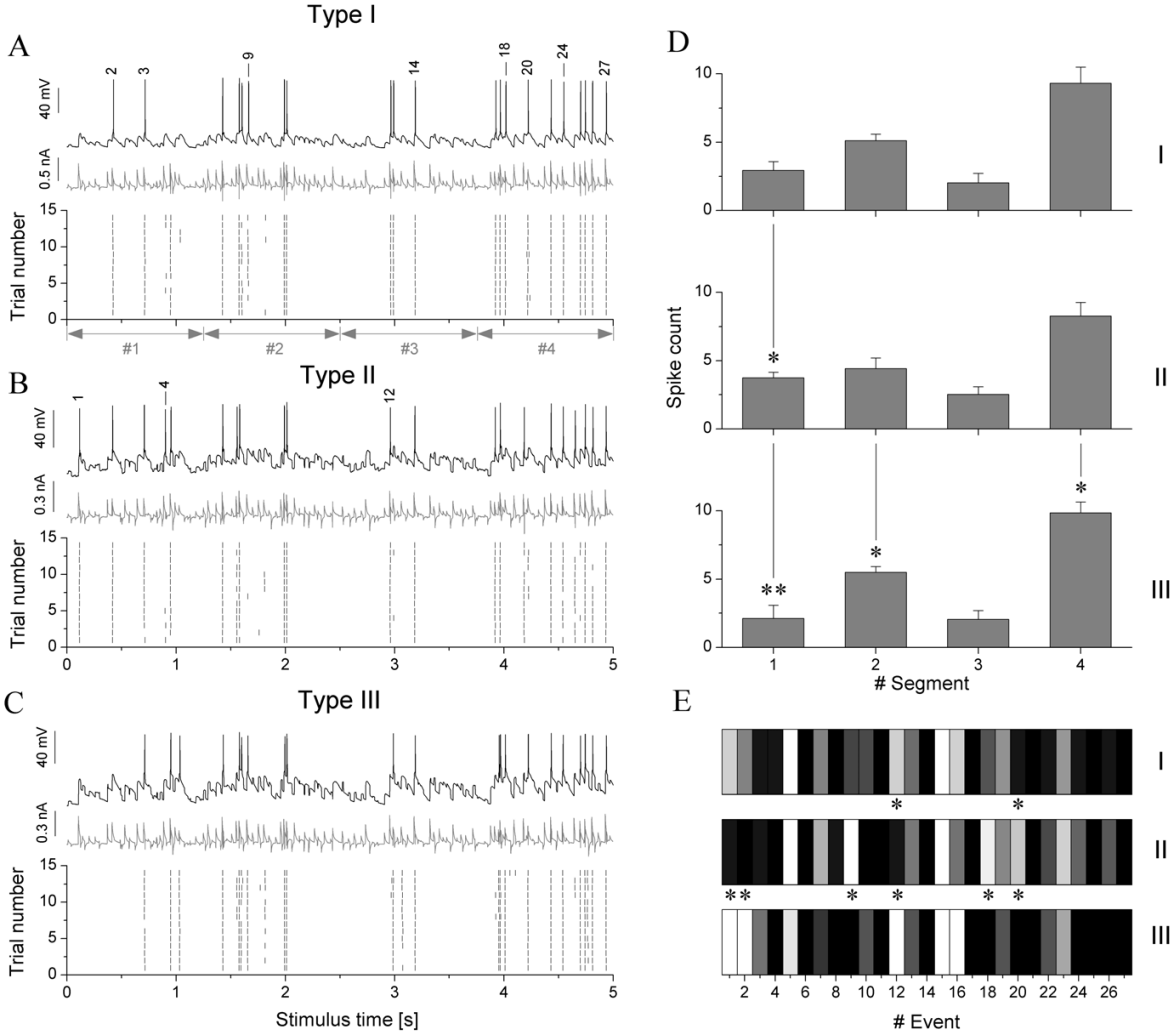
Different types of jcBNST neurons produce qualitatively similar firing patterns in response to identical synaptic inputs but display stronger differences at the single spike resolution. Type I, II and III neurons received the same 30 Hz noisy synaptic input and emitted spike trains depicted in A, B and C, respectively (voltage output, injected current and peri-stimulus scatter plots for each). Counting spikes in 4 equal and successive segments of the 5 s stimulus (1.25 s each, see gray arrows on A) we obtain the spike count bar graphs on D (mean±S.D., n = 6 for each cell type). Cell type III appears to fire less spikes in the first segment than cell type II and the relationship is reversed in segment #4. Significant differences are indicated by single or double asterisks (p<0.05 and p<0.01). Panel E shows the analysis of the fine structure of firing. Spike events in 27 locations along the stimulus are compared for the three cell types. Firing probability in the particular location is indicated by grayscale, white for p<0.2 and black for p>0.9 (linear grayscale between). Asterisks mark specific spike events, where firing probability is significantly different between cell types I and II or II and III. Some of the corresponding spikes events are labeled in A and B (see numbers 1–27 above spikes).

The experiments with gradually increasing synaptic inputs described above provided information on the conductance dependence of the spike number and precision of spike timing. When the target number of spikes was achieved, we repeated the stimulation with fixed maximal conductances for the three synaptic inputs. Due to the different biophysical properties of the three main types of jcBNST neurons, i.e. differential expression of their specific voltage gated membrane conductances, we expected that they would produce different spike responses to the same temporal pattern of synaptic inputs. However, as shown in Fig. 4, most spikes appear in the same locations for the three types of neurons. In fact, qualitatively the spike responses appear very similar for the three neuron types. Well reproduced features of the spike responses, for instance, are the doublets at t = 2 s of stimulus time, the long spike-free period between t = 2 and 3 s and the relatively intense firing after 4 s. These features are very similar across the entire spectrum of cells when this particular input (30 Hz noisy pattern) is presented. Using different voltage waveforms as presynaptic inputs results in different firing responses, but again, they are well reproduced across different cells (20–200 Hz frequency range as well as Poisson-trains, not shown). One might conclude that the synaptic input appears to have a stronger role in determining jcBNST spike emissions than the biophysical character of the neurons.

This view was further strengthened by pairwise comparison of the spike responses using similarity matrices. As earlier studies have shown, various implementations of spike train distance (analogous to similarity) perform well in distinguishing between neuronal responses or classifying cell types [15], [16]. In our analysis we used two methods for calculating pairwise similarities each offering its advantages. In the first case we started the procedure by calculating PSDFs for the neurons receiving the same frozen noise stimulus (30 Hz template). Then normalized spike train distances were obtained using the formula
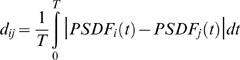
where *T* is the duration of the stimulus (5 s). This method is sensitive to both the existence and precise alignment of spike events in particular locations of the stimulus. Note that the shape of a particular peak in the PSDF depends on the number of observed spikes in that particular event (reliability) as well as their spike jitter (precision). Similarity is at maximum when peaks in the PSDFs of both neurons appear in the same locations and with similar shape. The second method only examines whether peaks in particular locations appear in the PSDF (amplitude and precise alignment is not taken into account). Whenever a reliable spike event is triggered by the same EPSP (same location) in both neurons, two peaks in the corresponding PSDFs will appear in identical locations and the total number of such occurrences is counted. The similarity is maximal when all 20 peaks appear in the same locations. Here, spike train distance is simply the count of matches subtracted from 20.

**Figure 5:**
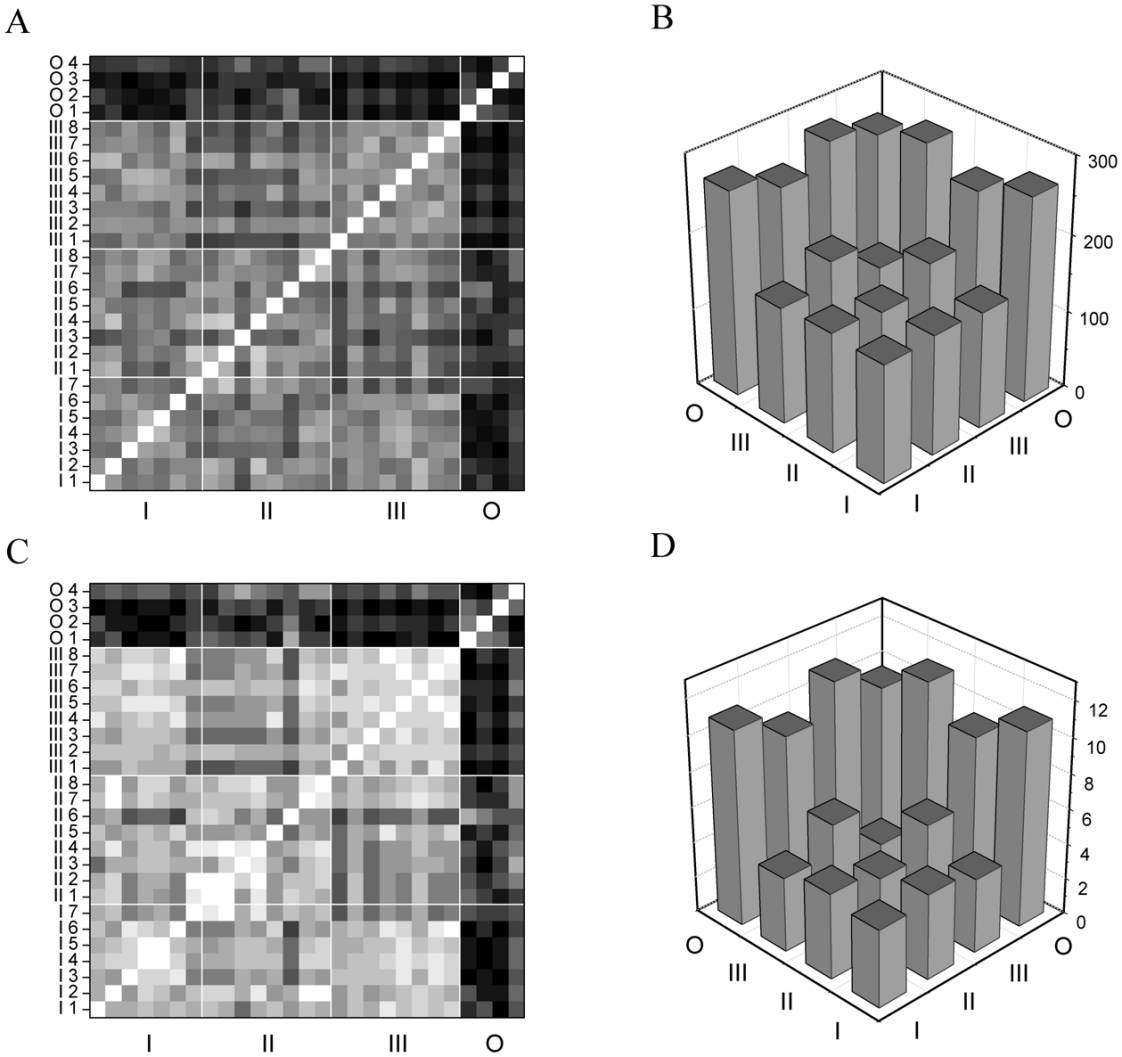
Similarity analysis of spike responses of jcBNST neurons reveals slight differences across cell types. Grayscale-coded matrices demonstrate pairwise spike train distances calculated from the responses of 27 neurons. The neurons were grouped into four clusters (types I, II, III and O for oscillatory) as defined by their responses to rectangular current stimulation. Panel A shows pairwise distances obtained from the absolute differences between the PSDFs (method 1). C is the result of the peak match counting (method 2). Average pairwise distances within groups and between groups are not significantly different, however, cell type III neurons within the group appear to produce more similar spike responses than others (III/III field is the brightest). Also, the average distance between type II and III neurons exceeds those between the I/II or I/III types. Panels B and D display the average distance values within the main partitions of the matrices (line of identity not included). The new oscillatory type neurons display firing patterns that are dramatically different from those of the other types of neurons.

Using the two methods for calculating the spike train distances we built matrices of the pairwise data. Figure 5 shows the results of such calculations. Type I, II and III neurons are grouped separately so within-group and between-group comparison can be quickly achieved. The grayscale maps show that pairwise similarities of spike responses between cell type groups are not markedly different from those within groups. Cell type III appears the most consistent across experiments in the sense that neurons within this group display a higher degree of similarity than the other types as indicated by the lighter gray within the III/III field. At the same time, responses of cell type II neurons show greater distances from cell type III and in a lesser degree, from cell type I responses, as indicated by the relatively darker parts of the similarity matrices (I/II and III/II fields). The two similarity maps also contain data from a novel cell type (type O for oscillatory) that are qualitatively very different from the other neurons responses. In fact, we find the highest distance values when responses of type O cells are compared to those from the other 3 types of neurons. These cells are described in detail later.

While the above analysis showed that spike responses from the 3 originally described biophysically different types of neurons are generally similar, we decided to have a closer look at the fine structure of firing so our analysis might reveal more subtle differences that are hidden in a qualitative assessment or when calculating the above gross measures of similarity. At this level of analysis we managed to identify several features and temporal parameters that were significantly different between but not significantly different within groups. One of such features that distinguish type II neurons from the rest is that they tend to fire more spikes in the beginning of the stimulus than the other types of neurons (compare Fig. 4B to A and C). Type III neurons, on the other hand, fire more spikes in the last part of the stimulus (after t = 3.5 s) with short interspike intervals being common here (Fig. 4C). Indeed, spike counts in four consecutive sections of the responses clearly demonstrate the differences between the cell types (see Fig. 4D). This comparison shows that type II and III neurons differ stronger than type I and III neurons do and this is in agreement with the visual assessment of the similarity matrices. We note that the statistical properties of the excitatory and inhibitory inputs were constant when designing the template waveforms, however, there are sections when the excitation is by chance stronger than the inhibition, and vice versa. Hence, we can identify sections - four in our example - where the overall synaptic input is qualitatively different from the others.

Finally we compared the relative occurrence of spike events in particular locations (specific EPSPs) of the stimulus for the three cell types. Evaluating the peri-stimulus density functions of 18 neurons (3 types, 6 neurons of each) we identified a total of 27 possible locations where marked peaks could be found. Although the cells were stimulated in a way that they produced in average 20 spikes per trial, the spikes did not always appear in the same locations among cells, hence the number for possible spike locations is greater than 20. In the following, we counted the number of trials where we did observe spikes at the 27 pre-selected locations for different type of neurons. Dividing this count by the total number of trials we obtained pooled event probabilities for the three types of neurons (Fig. 4E). This analysis showed that only a small percentage of spike events accounted for the bulk of all differences between the three neuron types. A grayscale map of event probabilities shows that most of the observed spikes are emitted in the same locations of the stimulus independent from the cell type, however, there are a few locations, “bits”, where the responses are markedly different. These locations are indicated with asterisks. For instance, type III neurons rarely fired in the beginning of the stimulation and location #1 and #2 (first two bits) see few spikes from this type of neurons. Type II neurons, however, fire reliably here, so the pooled spike event probability is high. Also, marked differences are seen at locations #9, #12, #18 and #20. Consequently, biophysical differences in the three types of jcBNST neurons lead to detectable differences only in a small percentage of events. Conversely, the majority of spikes (∼80%) are generated uniformly in most neurons despite their different biophysical characteristics. In this aspect these spikes can be considered as “trivial” events.

### A novel type of neurons with oscillatory properties

**Figure 6:**
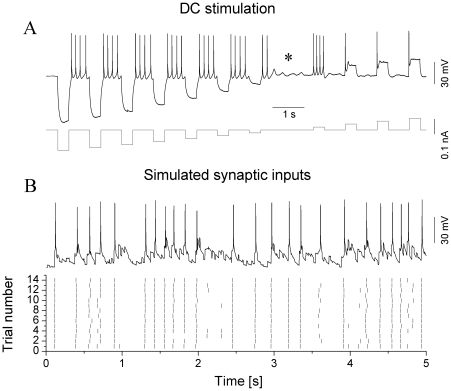
A novel type of neuron in the jcBNST displays oscillatory properties. A shows the effect of hyperpolarizing and depolarizing current steps (+20 pA increment). While this neuron displays post-inhibitory rebound firing like type II neurons, it maintains repetitive firing rather than emitting a short PIR burst. Intrinsic oscillation is apparent after releasing it from −20 pA hyperpolarization (asterisk). (B) The spike response of this neuron under the standard 30 Hz stimulus is fairly regular and appears very different from that of other type of neurons (compare to Fig. 4A–C).

Cell type II neurons are relatively easy to identify because of their prominent sag-current in response to moderate hyperpolarization with a DC pulse and because they fire spikes when released from hyperpolarization. Such post-inhibitory rebound is very characteristic and robust in cell type II neurons while absent in types I and III neurons. In a small number of cells we found a sag-current associated with a post-inhibitory rebound, which, however, lacked the burst appearance of typical type II neurons and was instead more protracted firing (Fig. 6A). Specifically, these cells produced near theta-frequency tonic firing (5–8 Hz) rather than a short burst after released from hyperpolarization. Furthermore, the spikes appeared to follow the time course of an endogenous membrane oscillation that usually dampened after a few cycles and the neuron ceased firing (Fig. 6A, asterisk). Another interesting feature of this type of neuron is that they fire only a single spike in response to maintained DC depolarization (+40 pA and higher). This indicates a biophysical mechanism for prevention of recurrent spiking under depolarization. Again, in this aspect they are markedly different from the other known cell types, including type II neurons. Considering the above findings these neurons might represent a novel cell type rather than being anomalous type II neurons. Remarkably, spike responses of these neurons under dynamic clamp stimulation were markedly different from the 3 previously described jcBNST cell types. We grouped these oscillatory type neurons together and calculated spike train distances from their responses as we did for the other three types of neurons. The grayscale similarity matrices shown in Fig. 5 demonstrate that such neurons produced firing responses that were vastly different from those of type I, II and III neurons. Besides, this group was found to be less homogenous than those of the standard cell types, especially type III, because within group similarities were only slightly higher than between group similarities. Inspecting the fine structure of spike responses of such oscillatory neurons we found that they tend to maintain a more tonic and steady firing than the other cell types under the stimulation with 30 Hz synaptic input (Fig. 6B). Indeed, strong spike events are found in locations where the other well known cell types produce no spikes at all (e.g. from t = 2 to 3 s of stimulus time). Besides, the firing patterns of the oscillatory neurons lack short interspike intervals like those found in the responses of cell type III neurons (ISIs as shorts as 10 ms). While the reliability and precision of spike events along the stimulus were not significantly different from those in the other type of neurons, the oscillatory cells transform the 30 Hz synaptic input into a very different firing pattern than the type I–III neurons. Qualitatively, the spike responses of the type O neurons are more regular and interspike intervals are more even than in the other cell types. As we have shown for the known cell types (I, II and III), the majority of their spikes were trivial in the sense that they reflected more the dynamics of the synaptic input than the biophysical character of the neuron. In the latter group of oscillatory neurons we find that most of the spike events are non-trivial because they have weak overlap with the responses of the other cell types. In fact, the main effect of the synaptic input is the initiation of a relatively stable and regular firing reminiscent to pacemaker activity.

### Diversity of spike dynamics at the millisecond scale

The results we obtained with dynamic clamp stimulation on various types of jcBNST neurons lead to interesting consequences. Conventionally, neurons in a wide range of nervous systems are classified into different physiological types using simple protocols of rectangular current steps. As we observed, jcBNST neurons classified as type I, II or III cells and displaying clear differences in their voltage output under DC stimulation, do not markedly differ when stimulated with stochastic inputs via dynamic clamp. Conversely, the latter approach identified a novel oscillatory type of neuron that produced markedly different spike responses despite resembling type II neurons under DC current stimulation. If we choose to focus on the very obvious physiological (or dynamical) differences between jcBNST neurons, we can identify three main cell types using the DC step method and only two cell types using the synaptic stimulation via dynamic clamp. One could say, cell type classification might depend on the stimulus protocol the experimenter happens to choose.

The fact that differences between the neurons' firing were detected only in a small percentage of spikes suggests that they integrate their synaptic inputs in a fairly similar manner (except the type O neurons). Nevertheless, biophysical diversity of neurons - even of the same - type has been demonstrated in a number of brain areas. The diversity is caused by the differential expression of specific voltage-gated membrane conductances and random cell-to-cell variations on the passive membrane properties of the neurons. We assumed that such random variations in the biophysical properties of neurons would have an impact on their firing responses in a way that appeared as random, within-group variations in one of the temporal parameters yet to be determined. To test this hypothesis we performed a thorough analysis of the fine structure of the spike responses.

**Figure 7:**
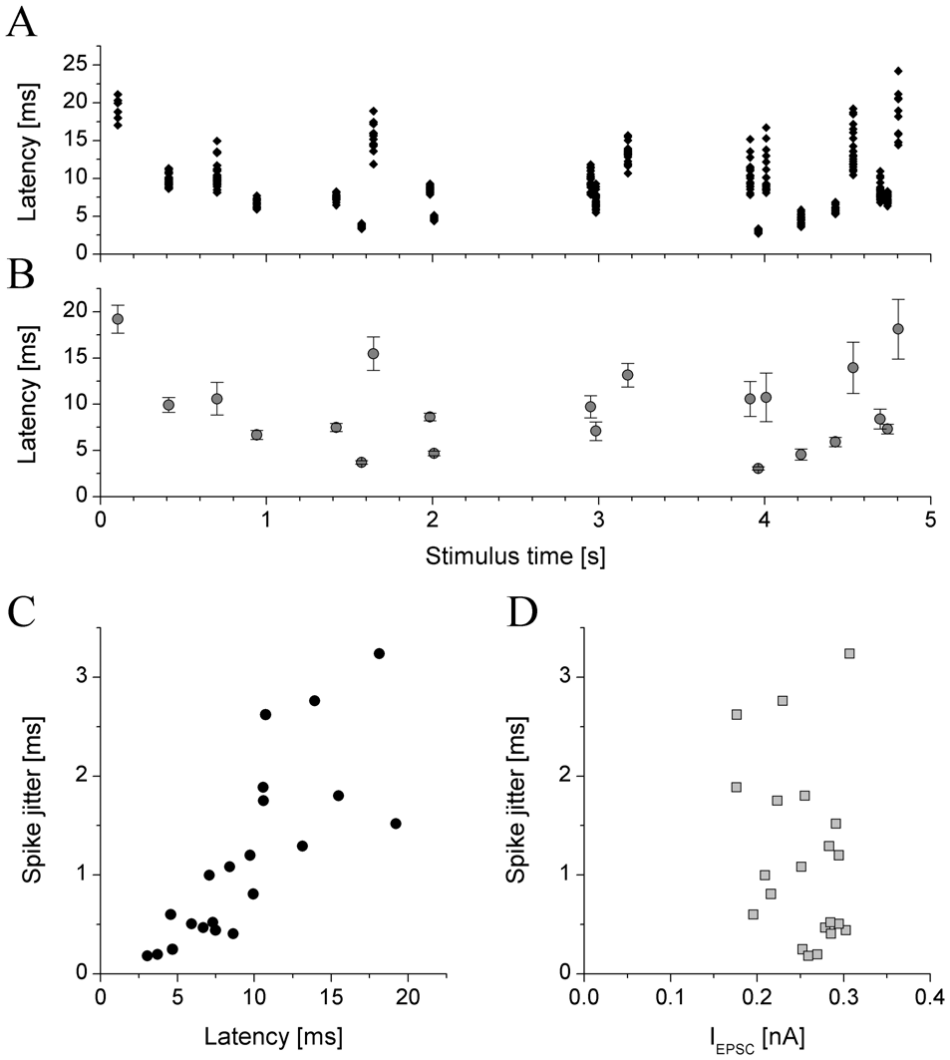
Analysis of pre- and postsynaptic spike timing in a type II neuron reveals strong correlation between latency and spike jitter. Latencies for each spike event are plotted against the stimulus time in A. Here, approximately 20 spikes were emitted in every trials. Means and S.D.s of the latencies were calculated and plotted in B. Latency values for specific locations of the input are well reproduced across trials, shown by the small S.D.s (same as spike jitter in per-stimulus plots). Spike latency and spike jitter are strongly correlated and the relationship is close to linear (C). At the same time, we find no correlation between the spike jitter and the amplitude of EPSC just preceding the postsynaptic spike (D).

As a general strategy, the spike responses of neurons are to be analyzed in relation to the input that was presented to the neuron and processed into a specific output firing pattern. Therefore, we analyzed the temporal relationship between the local synaptic conductance transients and the corresponding spikes in the receiving neuron. As described, the input coupled to the dynamic clamp consisted of excitatory and inhibitory waveforms containing “spikes” of variable amplitude, hence the jcBNST neurons received EPSCs and IPSCs of variable amplitude. Due to the small amount of overlap between the EPSCs it was always possible to determine which excitatory transient was the triggering event for any spike in the jcBNST neuron, hence we were able to measure pre- and postsynaptic spike latency for each event in the peri-stimulus plot (Fig. 7A). These latencies were distributed in a wide range, from 2 to 20 ms for most neurons. However, latencies in particular locations of the stimulus are well reproduced across trials as shown by their low spread (S.D.). Indeed, the standard deviation – identical to spike jitter of peri-stimulus scatter plots – is a small fraction of the local latency. The coefficient of variation, defined as S.D. divided by the mean is 0.1 for events with 10 ms latency and even less with shorter latencies. The relationship between the mean spike latency and its standard deviation is illustrated in Fig. 7C. This graph reveals a tight, positive correlation between these measures, a general behavior that is well reproduced among all types of neurons in the jcBNST. The slope of the regression line and the scattering of points varies from cell to cell, but the overall behavior is always discernable. Reasonably, one would expect that perhaps the most important factor setting the value for pre-postsynaptic latency is the strength of the synaptic input that triggers the spike emission. Clearly, a stronger EPSP would bring the postsynaptic neuron above firing threshold faster than a weaker EPSP. This is, indeed, clearly shown by the analysis of firing under gradually increasing EPSCs (Fig. 3). Due to the positive correlation between latency and spike jitter (Fig. 7C) one would then expect a clear, negative-slope relationship between the EPSC amplitude and the jitter. However, when examining the relationship between the amplitude of local EPSCs and the latency of their corresponding spikes, we find virtually no correlation (Fig. 7D). Hence, when synaptic inputs arrive to the postsynaptic cell continuously and in a temporally complex pattern, local EPSP strength is not the determining factor setting the latency of the postsynaptic spike. Seemingly, this contradicts the findings shown in Fig. 3. However, note that the accurate exponential relationship is observed for individual spike events where the synaptic strength is gradually increased. When spike events are compared among different EPSPs in a temporally complex stimulus, a different exponential relationship can be found for each EPSP-spike coupling. Latencies for event A and B can be very different even if the amplitudes of the triggering EPSPs were identical.

If latencies are so well reproduced in the responses of single neurons receiving the noisy synaptic inputs (low coefficient of variation), are the latency maps similar across neurons of the same type? This comparison is possible mostly because the neurons tend to fire in response to the same EPSPs, i.e. in similar locations of the stimulus waveform. As we observed, identical type of neurons fire spike patterns that overlap in 80 or higher percentage of the spikes. For example, a spike latency at t = 0.9405 s can be compared for neuron A and B, because they both fire in that particular location. Somewhat unexpectedly, we find no two similar latency maps when comparing these diagrams for type I, II or III neurons. In fact, each neuron produces a unique pattern of latencies in these diagrams, hence they are similar only to themselves. As noted, the low C.V. of latencies indicates that the neuron reproduces the same spike event with remarkable precision in the successive trials, but another neuron, even within the same type of cells, will fire in response to the same input with a different latency. A simple explanation for this finding would be that neuron A tends to fire with a latency that is, on average, a constant percentage (e.g. 50%) of that of the latency of neuron B. In this case, rescaling the latency map of neuron A would result in a map that nicely overlaps with that of neuron B. However, some spike events have a shorter latency in neuron A than in B but others have the reversed relationship. Hence, the simple linear rescaling does not work and the latency maps are found to be distinctive for every neuron. To verify our interpretation on the qualitative differences in the latency diagrams, we decided to perform a systematic pairwise comparison of the spike latencies between neurons. Again, we selected time series that contained approximately 20 spikes per trial (30 Hz tonic stimulus) and which were recorded from neurons that were clearly identified as type I, II or III cells according to their voltage responses to DC stimuli. Then we calculated latency values and their variance for spike events that were present in the spike responses of both neurons to be compared (i.e. spikes at identical locations). Since the spike jitter (variance of the latency) was heterogeneous for different neurons, the use of Welch-statistics was justified. A total of 3138 t-tests were performed (including 21 neurons) and significant differences were found in 83% of the pairs (p = 0.05). Within group comparison revealed significant differences in 75, 80 and 88% of pairs for type I, II and III neurons, respectively. In this respect, the fine structure of firing revealed substantial variations across the population of neurons even if they are classified as the same cell types. At the millisecond time scale and when considering the dynamics of EPSP-spike coupling, every neuron appears to process synaptic inputs in a way that is different from the rest.

## Discussion

Our experiments revealed several intriguing features of synaptic processing in the neurons of the juxtacapsular bed nucleus of stria terminalis. Our first finding was that biophysically distinct types of neurons as determined by conventional means of classification did not produce markedly different firing responses when stimulated with physiologically realistic synaptic conductance waveforms under dynamic clamp. Although the qualitative features and gross temporal parameters of the spike responses were similar across cell types, we observed significant differences in the fine structure of the firing, i.e. at the single spike resolution. In addition to the known three cell types, we identified a new type of jcBNST neuron with intrinsic oscillatory properties that displayed spike dynamics markedly different from the others. Finally, we showed that spike latency maps of neurons even from the same groups display great variations most likely due to the diversity of their intrinsic biophysical properties.

### Conventional vs. realistic stimulation of single neurons

According to the conventional view, the voltage output of synaptically isolated neurons under DC current stimulation reveals several important physiological properties that might suggest some feature of their operation in the intact brain. The voltage output in response to current steps, on the other hand depend on the intrinsic biophysical properties of the neurons such as the abundance or lack of specific voltage-gated ionic conductances. Indeed, this quick and reliable method has been commonly used to assess the neurons' overall physiological properties and to classify them into distinct cell types [4], [14], [17], [18]. When observing the behavior of the neuron under DC stimulation, one can identify several telltale signs of specific voltage-gated conductances that shape the voltage output. The slope of depolarization leading to the first spike, the sag-response under negative current injection or the post-inhibitory rebound are often considered as physiological correlates of important membrane conductances, especially when voltage clamp experiments confirm their existence.

Considering the wide repertoire of biophysical properties that mammalian neurons display one can expect correspondingly rich behavior in their dynamics. While computational models of neurons have been of great value in such investigations, biological experiments with physiologically realistic inputs are to be performed to gain a clearer understanding on how these neurons might work in the active synaptic environment of the intact brain. The dynamic clamp technique offers a great opportunity for such investigations [7], [18], [19]. When repetitively stimulating the neurons using the same random conductance waveforms as artificial synaptic inputs, one can assess the reliability and precision of their firing [20], [21], [22]. These parameters are considered as quantitative measures of the neurons performance in the processing of synaptic inputs. Admittedly, *exact* repetitions of synaptic messages such as those in the frozen noise experiments are unlikely in the natural conditions. On the other hand, a population of presynaptic neurons can synchronously deliver inputs to multiple postsynaptic targets often with different biophysical character. Our experiments were designed to mimic these very conditions. It is also notable that a single sweep of the noisy stimulus template (5 s length) is actually consisted of hundreds of excitatory and inhibitory conductance transients, therefore the stimulated neuron experiences a rich and variable input unlike in experiments with DC current injection.

The 30 Hz template waveform we used the most frequently in our experiments can be considered as a way to simulate a moderate intensity synaptic input, but we did not aim to simulate strong synaptic bombardment that is characteristic of highly active circuits in the awake brain [7]. Our stimulus was, however, well-suited to study the impact of single EPSPs on postsynaptic activity and to assess how the biophysical character of the neuron affected the temporal pattern, reliability and precision of spike responses. In our experiments the potency/impact of each EPSP could be quantified, pre- and postsynaptic spike latencies could be accurately measured.

### Distinct cell types can function in a qualitatively similar manner under synaptic input

The jcBNST neurons of our study have been characterized using conventional DC current stimulation as well as voltage clamp [14]. The three cell types contribute to the majority of the jcBNST neurons and they have been classified as medium-sized spiny neurons according to their morphological properties [23]. Our observations on the voltage output of such neurons in response to hyperpolarizing and depolarizing current injections were in good agreement with those in the Hammack study. The authors of this work identified five important voltage-gated ionic currents that are differentially expressed in the cell types and each of those has a strong impact on the voltage output of the neurons under DC stimulation. These are the hyperpolarization-activated nonspecific cation current (I_h_), the low-threshold Ca-current (I_T_), the fast transient K-current (I_A_), the inwardly rectifying K-current (I_K(IR)_) and the persistent Na-current (I_NaP_). Clearly, these are ubiquitous in neurons of other brain areas, too, hence the jcBNST neurons offer a good experimental subject to study how the neurons integrative/computational properties depend on their biophysical character.

Our experiments showed that neurons with different biophysical properties can produce rather similar spike output in response to the same synaptic input. Indeed, type I, II and III neurons as classified by the conventional methods produce apparently different voltage output in response to DC current steps, but they can produce similar firing patterns when receiving the synaptic input under dynamic clamp. One would expect, the differential activation and deactivation profile of voltage-gated conductances in the three types of neurons will eventually result in deviations in their spike responses, i.e. they will follow different trajectories in their phase spaces. However, we observed that the majority of the spikes emitted by the three types of neurons occur in the same locations of the input waveform. Our preliminary calculations also showed that these “trivial” spikes can be well reproduced in a simple leaky integrate-and-fire model that is presented the same input as the biological neurons. In this respect, the trivial spikes are input driven events that occur with high probability and independently from the intrinsic biophysical properties of the neuron. As previously showed, the three types of jcBNST neurons differ significantly in the relative magnitude of their I_h_, their low-threshold Ca-currents and their inwardly rectifying K-currents [14]. Our results suggest that even strong differences in the magnitude of these conductances will not cause qualitatively different firing output in the neurons. Considering the low voltage threshold of activation for many of these conductances one can envision that they play a relatively minor role in shaping the ongoing firing patterns of neurons when they are relatively depolarized and bombarded by excitatory inputs. In agreement with this notion, qualitatively similar firing patterns have been demonstrated in pyramidal neurons with markedly different amount of I_h_ currents [24]. Nevertheless, qualitatively similar responses - as interpreted by the experimenter - do not mean that the three types of neurons function the same way under identical synaptic inputs. Indeed, a small percentage of spikes, the “non-trivial” ones are reliably emitted by one type of neurons but not the other types. Typically, these are the spikes where the integrate-and fire model fails to reproduce the dynamics. We suggest that these spike events correspond to the specific conditions of synaptic input interacting with the intrinsic properties of the neuron when the biophysical differences grow to the detectable level. Such conditions can occur during the summation of excitatory postsynaptic potentials [24]. If 80–90% of spikes are reproduced consistently in the three types of neurons, does the smaller fraction of non-trivial spikes matter? Clearly, if neurons are considered as rate-coders, then a small percentage of “missed” spikes will probably not make much of a difference and the downstream populations of neurons will not detect the differences in the output patterns. However, synchronization of spatially distinct groups of neurons or fast sensory processing requires temporally precise regulation of spiking and the impact of non-trivial spikes can be significant in such processes. Also, forms of short-term synaptic plasticity such as depression or facilitation especially when coupled with resonant properties of the postsynaptic neuron can result in high sensitivity to the temporal structure of the input spike pattern [25]. In such systems the presence or lack of even single spikes in the input can lead to markedly different output from the postsynaptic cell.

As part of our study, we identified a novel cell type of oscillatory neurons. Analyzing the behavior of this new type of neurons we realize that in some cases neurons do produce very different output in response to the same synaptic input. While the previously identified three types of neurons produce qualitatively similar patterns, this new type follows a very different trajectory. One of the intriguing features of its firing is that the interspike intervals display far less variations than those in the other 3 types of cells. Apparently, the newly identified oscillatory neurons tend to fire tonically in the presence of synaptic input and single EPSPs and IPSPs play a modulatory rather than a driving role in spike emissions. As we observed, this neuron type can fire tonically and persistently after the termination of a strong inhibition (DC pulse). This pacemaker type activity might be initiated by the synaptic inputs as delivered by the dynamic clamp. Hence, under such conditions the dynamics of the oscillatory type cells is not governed by the input but equally or even more shaped by their intrinsic properties. The specific voltage-gated conductances that might be responsible for the intrinsic oscillation and regenerative spiking in such cells are yet to be determined.

### Consistency and diversity of firing

The three types of neurons reproduced firing patterns with high reliability and precision when stimulated with a stochastic synaptic input via dynamic clamp. As we showed, the majority of spikes were emitted in the same locations of the stimulus, therefore, spike responses were consistent across neurons from different groups. At the level of single spikes we found another very consistent behavior, namely the exponential dependence of spike timing on the magnitude of the excitatory input [21]. When the total conductance gain of the synaptic input was incremented in small steps, spikes in specific locations were emitted with gradually decreasing latency, and the relationship between the conductance and latency was accurately fitted with a monoexponential. In this respect, the latency of single spikes even in complex firing patterns could be well predicted if the amplitude of the preceding excitatory conductance transient (or its EPSC) is known. However, this is not feasible for several reasons. When spikes are produced in response to temporally complex conductance inputs, each spike latency will depend on the local EPSC and the preceding history of the postsynaptic membrane potential. Although gradual increase of the total synaptic gain will result in exponential decrease of individual spike latencies, they will follow different paths, so there is no one-fits-all relationship that can be applied to every spike events. The other problem is the interference between adjacent spikes. Refractoriness and the activation of potent outward currents following a spike can delay the emission of the next one. These findings show why it becomes increasingly more difficult to predict the latency – and precision – of a spike event when it is embedded in an intense barrage of excitatory synaptic inputs. When we compare latency maps of different neurons, the picture becomes even more colorful. Notably, even using identical types of neurons and after careful normalization of stimulation conditions, we find no two firing patterns that contain statistically matching latency pairs for specific locations of the stimulus. At this level we can conclude that all neurons behave differently from the others and they will fire spikes with different latencies in response to synchronous synaptic excitation. The mismatch between the timings of postsynaptic spikes can be several milliseconds, a rather significant dispersion and presenting a potential problem for explaining precise synchronization in further downstream populations of neurons. However, convergence of many, temporally distributed excitatory inputs have been shown to be effective in reducing postsynaptic spike jitter [21], so precise synchronization can be maintained by appropriate synaptic topology. Additionally, inhibitory feedback might be also effective in reducing the temporal spread of spikes as they are transmitted through different stages of processing [26].

In conclusion, our results show that neuronal types with distinct biophysical properties can produce similar spike patterns in response to the same complex synaptic input. However, the degree of similarity depends on the time scale that is chosen to analyze their responses. At the longer time scale neuronal responses in the three previously described neuronal cell types appear similar, so this analysis does not distinguish them. A higher resolution analysis at shorter time scales reveals the existence of non-trivial spikes that show consistent variations among the three previously described neuronal cell types. A great degree of diversity among neurons independently of their distinct biophysical properties is observed at the highest resolution (ms). Therefore, the biophysical properties of neurons as revealed by conventional DC stimulation protocols are not performing well in predicting their responses to complex synaptic stimulation. Thus, caution should be taken when extrapolating results with conventional stimulation to the functional properties of neurons in microcircuits or higher levels of organization in the nervous systems.

## Materials and Methods

### Brain slices and electrophysiology

All experimental protocols were consistent with guidelines issued by the National Institutes of Health and approved by our Institutional Animal Care and Use Committee (protocol number 07-0068). Acute brain slices were prepared as previously described [11], [27] with minor modifications. Briefly, coronal rat brain slices (350 µm) were collected from the rostral cerebrum of Wistar rats using a Capden vibrating microtome (Loughborough, England) in oxygenated artificial cerebrospinal fluid (ACSF) consisting of (in mM) 130 NaCl, 3.5 KCl, 24 NaHCO_3_, 1.25 NaH_2_PO_4_, 2.2 CaCl_2_, 10 d-glucose, and 2 MgSO_4_, pH 7.4. Slices were preincubated in ACSF for 1 hour at 32°C and then maintained at room temperature for at least 30 min before being transferred to a submerged recording chamber at 31°C.

Slices of brain tissue containing the BNST were placed in a superfusion chamber and visualized with a Leica stereomicroscope under low magnification. Single neurons were not visualized during electrode insertion and the experimental session (blind recordings). Intracellular current clamp and dynamic clamp experiments were performed in whole-cell configuration using 10–12 MΩ patch pipettes filled with intracellular solution containing (in mM): KMeSO_4_ 120, KCl 10, MgCl_2_ 3, HEPES 10, Phosphocreatine 10, MgATP 2, GTP 0.2; osmolarity set to 280–290 mOsm, pH 7.2. Synaptic isolation of jcBNST neurons was achieved by blocking glutamate and GABA receptors using 10 µM 6-cyano-7-nitroquinoxaline-2,3-dione (CNQX), 50 µM AP-5 and 30 µM bicuculline in the bath.

Recordings and intracellular stimulation were made using a Multiclamp 700 amplifier (Axon Instruments) in the bridge mode. Stimulus waveforms (both rectangular and spike-like) were generated using the data acquisition software DASYLab 6.0 (Dasytec, Amherst, NH) in a Windows computer equipped with a National Instruments PCI-MIO-16-E4 multifunctional board. We used standard rectangular current commands for conventional physiological characterization of the jcBNST neurons. Specifically, we delivered 350 ms pulses of intracellular current incremented by 20 pA from −200 pA to +100 pA or higher levels, depending on the cell type.

### Dynamic clamp

We elicited firing activity in the jcBNST neurons by injecting them with simulated excitatory and inhibitory synaptic inputs via dynamic clamp (Fig. 2). To achieve this, we first generated artificial presynaptic voltage waveforms resembling random firing activity in populations of excitatory and inhibitory neurons. These analog waveforms (templates) consisted of 5 ms wide spike-shaped voltage transients that departed from and returned to a rest state of −60 mV. In order to induce variable amplitude synaptic currents in the postsynaptic biological neurons, we also introduced amplitude variation of the spike-shaped voltage transients such that their peak value ranged from −30 to 0 mV in a uniform distribution. In each experiment we used one excitatory and one inhibitory input that were designed using the same template parameters but uncorrelated otherwise. The excitatory and inhibitory voltage waveforms were coupled to the analog inputs of the dynamic clamp computer. Simulated chemical synaptic currents were computed using the formula
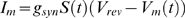
where *I_m_* is the postsynaptic current (injected into the jcBNST neuron), *g_syn_* is the maximal synaptic conductance, *V_rev_* is the synaptic reversal potential and *V_m_*(*t*) is the neuron's membrane potential. Transmitter release is modeled by an instantaneous activation term *S*(*t*) given by the differential equation
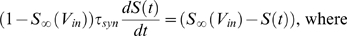


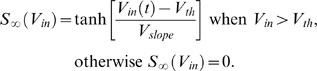

*V_in_* is the input voltage waveform (either the excitatory or the inhibitory voltage) and it serves as the presynaptic membrane potential for the dynamic clamp, 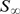 is the steady state synaptic activation, *τ_syn_* is the synaptic characteristic time constant, *V_th_* is the synaptic threshold voltage, and *V_slope_* is the synaptic slope parameter. The above parameters were set independently for the three synaptic conductances used in our experiments. The input from the excitatory voltage waveform (Fig. 2E, Exc) was used to evoke rapid (AMPA-type) and slow (NMDA type) excitatory postsynaptic potentials. The synaptic time constant (*τ_syn_*) was 10 and 50 ms for the AMPA- and NMDA-type connections, respectively and the reversal potential (*V_rev_*) was 0 mV for both. The second voltage waveform served as the GABAergic inhibitory input (Fig. 2E, Inh; *V_rev_* = −68 mV, *τ_syn_* = 10 ms). Excitatory inputs drove the firing, while the inhibitory input played a modulatory role in spike emissions.

The template waveforms and the postsynaptic voltage signal were connected to the analog inputs of a Digidata 1200B board that was the interface to the dynamic clamp software StdpC v. 1.9. [28]. This software can provide up to 6 independent inputs from simulated presynaptic neurons to the biological neuron. Additionally, it allows the experimenter to automatically change synaptic parameters such as maximal conductances in predetermined time points during the experimental trials. We used equal conductances for the two excitatory inputs (AMPA and NMDA type) and twice the conductance for the GABAergic input (e.g. 5/5/10 nS). The rationale for this setting was to keep excitation and inhibition balanced and proportional across experiments. Additionally, this setting assured that both types of inputs would exert their impact on the spike dynamics by differentially activating/deactivating intrinsic membrane conductances. Typically, the duration of the random presynaptic waveforms was 5 s and they were repeatedly presented every 13 s (frozen noise protocol), hence, the neurons were at rest for 8 s between stimuli. The two synthetic presynaptic voltage waveforms, the injected synaptic current and the voltage output of the biological neuron were acquired simultaneously at a 20 kHz sampling rate. To compare spike responses of the 3 jcBNST neuronal types we maintained the spike count constant (mostly 20) among different neurons by adjusting the 3 conductances while maintaining the aforementioned 1/1/2 ratio. The 3 maximal conductances were either changed manually until the targeted spike response was observed in two successive trials or, more frequently, they were automatically incremented by using the scripting feature of our software. Spike emissions in the stimulated neurons were detected on-line (by seeking local maxima of the derivative of membrane potential) and the arrival times were saved into ASCII files. We measured spike arrival times at 50 µs accuracy and in reference to the onset of the stimulus in each trial (sweep). The presynaptic voltage waveforms (Exc, Inh) were generated and the response of the jcBNST neuron was recorded by the DASYLab 6.0 program (Dasytec, Amherst, NH), hence, two separate computers were used for data acquisition and for the dynamic clamp (Fig. 2E).

### Data analysis

Firing patterns obtained in the dynamic clamp experiments were initially analyzed using peri-stimulus scatter plots. Reliable spike events in such plots manifested as vertically aligned tick marks, i.e. when a spike was emitted repeatedly in the same location of the stimulus. The statistical analysis was based on the evaluation of peri-stimulus density functions (PSDFs) constructed from the spike arrival times of the successive trials [29]. PSDFs were obtained by convolving the spike times with a unity-area Gaussian function called the kernel according to the formula:
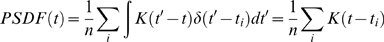
where δ(t) is the delta function, K(t) is the kernel with 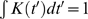, and n is the number of stimulus presentations. The PSDF is a function of time relative to the stimulus onset. The Gaussian-kernel based PSDF provides a smooth and accurate estimation of firing frequency along the time of the stimulus and excels over conventional peri-stimulus time histograms. Reliability for each spike event was calculated by counting the trials with successful spike emission and dividing this count by the total number of trials. When this ratio was below 33%, the event was considered as unreliable and not taken into account to obtain the *mean* reliability for the entire experiment. The rationale for discarding the low-reliability events is that such spikes often introduce small peaks in the PSDFs that can interfere with the detection of spike events. This type of pre-filtering removed only a small percentage of spikes. The mean reliability was calculated by simple averaging of single event reliabilities. The precision of spike timing was characterized by the temporal jitter of spikes within reproducible events. The standard deviation of spike times was calculated for each spike event in a peri-stimulus plot and the arithmetic mean of the individual S.D.s was calculated. The mean spike jitter served as a scalar measure of the overall spike timing precision in the experiment.
